# A Comparison of the Affectiva iMotions Facial Expression Analysis Software With EMG for Identifying Facial Expressions of Emotion

**DOI:** 10.3389/fpsyg.2020.00329

**Published:** 2020-02-28

**Authors:** Louisa Kulke, Dennis Feyerabend, Annekathrin Schacht

**Affiliations:** ^1^Affective Neuroscience and Psychophysiology Laboratory, University of Göttingen, Göttingen, Germany; ^2^Leibniz ScienceCampus Primate Cognition, Göttingen, Germany

**Keywords:** facial expressions of emotion, automatic recognition, EMG, emotion recognition software, affectiva

## Abstract

Human faces express emotions, informing others about their affective states. In order to measure expressions of emotion, facial Electromyography (EMG) has widely been used, requiring electrodes and technical equipment. More recently, emotion recognition software has been developed that detects emotions from video recordings of human faces. However, its validity and comparability to EMG measures is unclear. The aim of the current study was to compare the Affectiva Affdex emotion recognition software by iMotions with EMG measurements of the zygomaticus mayor and corrugator supercilii muscle, concerning its ability to identify happy, angry and neutral faces. Twenty participants imitated these facial expressions while videos and EMG were recorded. Happy and angry expressions were detected by both the software and by EMG above chance, while neutral expressions were more often falsely identified as negative by EMG compared to the software. Overall, EMG and software values correlated highly. In conclusion, Affectiva Affdex software can identify facial expressions and its results are comparable to EMG findings.

## Introduction

Identifying which emotion somebody is expressing is a crucial skill, facilitating social interactions. The human ability to express emotions in their faces has already been studied by Darwin (1872), and since then, ample research has focused on interpreting emotional expressions. Different theories have been built, ranging from the view that many different emotional expressions are possible (e.g. Scherer, 1999; Scherer et al., 2001; Ellsworth and Scherer, 2003) to the view that a limited number of distinct and basic categories of facial emotion expressions can be distinguished (Ekman, 1992, 1999). In a laboratory context, facial expressions of emotions can be measured using Electromyography (EMG, Fridlund and Cacioppo, 1986; Van Boxtel, 2010). EMG records muscle activity using electrodes placed on the skin surface. Importantly, distinct muscle activity is observable in response to different emotions. Specifically, the zygomaticus mayor muscle reliably becomes active during expressions of happiness or more generally joy (smiles). The corrugator supercilii muscle – the muscle that draws the eyebrow downward and medially and thus produces frowning – is related to particularly angry facial expressions (Dimberg, 1982; Tan et al., 2012), to negative non-facial stimuli (e.g. Bayer et al., 2010; Künecke et al., 2015) as well as cognitive processes of increased load (Schacht et al., 2009; Schacht et al., 2010). Activity of the corrugator and zygomaticus muscles can be used to dissociate between positive and negative emotions, with particularly corrugator activity clearly related to emotions of negative valence (Larsen et al., 2003; Tan et al., 2012). Therefore, many EMG studies focused on measuring the activity of these two muscles in response to pictures of happy and angry faces (Dimberg and Lundquist, 1990; Dimberg and Thunberg, 1998; Dimberg et al., 2000; Dimberg and Söderkvist, 2011; Otte et al., 2011; Hofman et al., 2012). For example, this technique has been used to study voluntarily controlled compared to automatic facial reactions to emotional stimuli (Dimberg et al., 2002), responses to unconsciously perceived emotional faces (Dimberg et al., 2000), effects of perceived fairness on mimicry (Hofman et al., 2012), and neural mechanisms involved in facial muscle reactions (Achaibou et al., 2008). Research also investigated individual differences in muscle responses, e.g. due to gender (Dimberg and Lundquist, 1990), empathy (Dimberg et al., 2011; Dimberg and Thunberg, 2012) and anxiety of individuals (Kret et al., 2013). In summary, EMG is an established method, widely used to study facial expressions of emotion.

An alternative, well established method for assessing facial expressions of emotion is the Facial Action Coding System (FACS), developed by Ekman and Friesen (1976). This anatomy-based system allows human coders to systematically analyze facial expressions for their emotional information based on the movements of 46 observable action units (Ekman and Friesen, 1976). While the system has been widely used over the last three decades, the applicability of the method has been limited by the requirement of certified coders and highly time-consuming nature of the coding processes. However, more recently, novel automatic software solutions have been developed, which represent promising tools in overcoming the limitations of human FACS coding through an easier applicability and faster processing of expressions recorded. These automatized approaches were found to be as reliable as human coding for emotion detection (Terzis et al., 2010; Taggart et al., 2016; Stöckli et al., 2018), while saving time (e.g. Bartlett et al., 2008).

Software includes e.g. EmoVu (Eyevis, 2013), FaceReader (N. I. Technology, 2007), FACET (iMotions, 2013), and Affectiva Affdex (iMotions, 2015). To investigate the suitability of this software for research, it needs to be verified whether it (i) reliably detects emotions and (ii) is comparable to previously established methods like EMG. The investigation of the performance of this new technology might benefit future research by establishing attractive alternative methods for the automatic detection of facially expressed emotion. For example, while EMG is widely used because of its mentioned benefits, it is restricted to laboratory settings, and applying electrodes on a participant’s face might not always be desirable for all kinds of experiments. Automatic software may help to overcome these limitations as only a camera for video recording is needed, which makes it easier for experimenters to investigate facial expressions under more natural conditions. The present study will focus on the Affectiva software, which classifies images and videos of facial expressions for the displayed emotions based on a frame-by-frame analysis. The reliability of the Affectiva software has already been confirmed in validation studies on static images (Stöckli et al., 2018) and videos (Taggart et al., 2016), that demonstrated reliable emotion recognition by the software. Its comparability with EMG remains, however, unclear. Other face emotion recognition software [FaceReader (D’Arcey, 2013) and FACET (Beringer et al., 2019)] has been validated by comparing the software computations for happy and angry expressions with EMG results. D’Arcey (2013) correlated FaceReader scores with EMG measures of the zygomaticus and the corrugator supercilii muscle to investigate comparability. Based on this approach, the aim of the current study was to compare the capability of the Affectiva software to appropriately classify emotions with EMG measurements of the zygomaticus mayor and the corrugator supercilii muscles. Note that Beringer et al. (2019) only measured expressions of people who were trained in the expression of happy and angry emotions. In contrast, the current study used a large sample of untrained participants, to ensure that more natural expressions were measured.

Participants are faster at producing facial expressions of emotions and show stronger activations of the responsible muscle groups if they view a face expressing the same emotion (Korb et al., 2010; Otte et al., 2011). Therefore, in the current study, participants were instructed to imitate a facial expression (happy, angry, neutral), while equivalent face stimuli were presented to them on a computer screen. These three expressions were chosen because they can be reliably measured with EMG (Dimberg and Lundquist, 1990; Dimberg and Thunberg, 1998; Dimberg et al., 2000; Dimberg and Söderkvist, 2011; Otte et al., 2011; Hofman et al., 2012). Muscle responses to emotional faces can arise as early as 300–400 ms after exposure (Dimberg and Thunberg, 1998), and therefore were measured for a 10-s period starting with the onset of the face presentation. As muscle responses have been shown to occur stronger on the left side of the face (Dimberg and Petterson, 2000), we recorded EMG from this side. Participants were asked to imitate facial expressions based on images from a validated face database, in order to create highly controlled facial expressions, related to a face database commonly used for research. However, it should be noted that this task neither required the person to feel the emotion nor necessarily triggered it and rather prioritized controlled expressions over natural and spontaneous emotional processes.

The current study investigated whether the Affectiva software can identify different facial expressions (smile, brow furrow) and the emotions related to them (termed in the software as “joy”^1^ and “anger” vs. neutral) as efficiently as EMG, by testing participants once with EMG and once with a video recording in separate sessions, with the latter analyzed off-line with Affectiva software.

We expected Affectiva to report a higher probability for a happy expression compared to a neutral or angry expression in the happy condition and a higher probability for an angry expression compared to a neutral or happy expression in the angry condition.

We further expected significant correlations between EMG and Affectiva measures. The study was preregistered with the Open Science Framework (doi: osf.io/75j9z) and all methods and analyses were conducted in line with the preregistration unless noted otherwise. In addition to these planned analyses we conducted an exploratory test to investigate whether EMG and the Affectiva software can be used simultaneously. For this, video recordings of the EMG condition were analyzed with the Affectiva software to investigate whether the software can reliably identify facial emotions when these are partially covered up with electrodes.

## Materials and Methods

### Participants

Twenty students between 18 and 29 years (mean age = 21 years, *SD* = 2.6, 17 female) from the University of Göttingen and the HAWK Göttingen participated in return for course credit. The sample size was based on previous research (Dimberg, 1982; Otte et al., 2011). All participants were right- handed, had normal or corrected to normal vision (only by contact lenses, no glasses) and no neurological or psychiatric disorders according to self-report. Five additional participants were tested but excluded due to not fulfilling the original inclusion criteria (2), technical failure (2), or because they did not complete the study (1).

### Task Design and Stimuli

Three types of facial expressions were recorded from the participants (happy, angry and neutral) using a video camera and EMG electrodes, respectively. The task was implemented in PsychoPy (Peirce et al., 2019) and consisted of two blocks, the order of which was counterbalanced. Facial expressions of participants were recorded with a C922 Pro Stream Webcam (Logitech, resolution: 1080 × 720 px, 30 frames per second) during both blocks. In addition, one of the blocks included a facial electromyogram (EMG) recording. Lighting was kept constant during recordings with one direct current light source illuminating the room from above. A sufficient video quality for the Affectiva analysis was achieved by averaging the Affectiva AFFDEX quality score for every participant and excluding participants with a mean score below 75%. Participants were asked to position their face between the two bars of an adjustable headrest to ensure a central position of the face during recordings; however, their heads were not constrained by the chinrest, but it rather served as an orientation for position. The experimental task for the participants was to imitate the emotion expressed on each of 60 frontal portrait pictures (20 per emotion category) selected from the Karolinska Directed Emotional Faces (KDEF) database (Lundqvist et al., 1998), with 10 female and 10 male faces in each condition (happy, angry, and neutral). Face pictures were presented in a randomized order. Images were edited to the same format with Adobe Photoshop CS6 by matching luminance across images and applying a gray mask rendering only the facial area visible. The task started with a short, written introduction after which the participants could proceed by pressing the space button. During each trial, a fixation cross was presented for 5 s, followed by the stimulus being presented for 10 s at the center of the screen. Participants could take a short break after a sequence of 20 stimuli had passed.

### iMotion Affectiva

Videos were recorded within intervals ranging from the onset to the offset of the stimulus (10 s). Videos were imported in iMotions Biometric Research Platform 6.2 software (iMotions, 2015), and analyzed using the Affectiva facial expression recognition engine. Note that due to an update this is a more recent software version, than originally preregistered. Emotion probabilities were exported for all 60 stimuli per subject.

### EMG Recording and Pre-processing

The EMG was recorded from 10 electrodes during one of the two blocks in order to measure zygomaticus mayor and corrugator supercilii activity [two electrodes per muscle, based on Fridlund and Cacioppo (1986), and additional reference electrodes, as described below]. All data were recorded with a Biosemi ActiveTwo AD-box at a sampling rate of 2048 Hz. The skin around electrode placement sites was cleaned with a soft peeling and an ethanol solution to enhance electrical contact between skin and electrode. Electrodes were prepared with electrode gel (Signagel) before the arrival of the participant. Electrodes were attached at the left side of the face using bipolar placement as suggested by Fridlund and Cacioppo (1986). For measuring *Zygomaticus major* activity, a line joining the *cheilion* and the *preauricular depression* was drawn with an eye pencil. The first electrode was placed midway along this line and the reference electrode was placed 1 cm inferior and medial to the first. To measure *corrugator supercilii* activity, an electrode was placed directly above the brow and the reference was affixed 1 cm lateral and slightly above the other electrode. As a precautionary measure, alternative reference electrodes were attached behind both ears above the mastoids but not considered for further analysis. The two ground electrodes (required for online display of channels) were placed 0.5 cm left and right from the midline directly below the hairline. Two electrodes were attached to the *Orbicularis oculi* (1 cm below right border of the eye and 0.5 cm inferior and lateral to the first) to measure eye blink artifacts.

Data was processed with Brain Vision Analyzer 2.1 (Brain Products GmbH, Munich Germany). The following steps were conducted for each subject separately: A high-pass filter at 20 Hz, a low-pass filter at 400 Hz and a notch filter at 50 Hz were applied. The *zygomaticus major* and *corrugator supercilii* channels were re-referenced to their respective reference electrode to remove common noise between bipolar channels. The resulting data was rectified and segmented into three emotion-specific segments (happy, angry, and neutral), which consisted of 20 epochs of 10.200 ms, respectively, starting 200 ms before stimulus onset. A baseline period from 200 ms before until stimulus onset was subtracted from each data point. Segments were averaged per subject and the mean amplitude between 0 and 10 s after target onset was exported.

### Procedure

This study was approved by the Ethics Committee of the Institute of Psychology at the University of Göttingen and conducted in accordance with the World Medical Association’s Declaration of Helsinki. After arrival, the participant was seated in an electromagnetic shielded chamber and provided written informed consent as well as relevant personal information. Participants were shortly briefed about the process of electrode attachment and then prepared for EMG recording directly before the EMG trial. After the EMG setup was complete, the electrode offset was checked and adjusted to below 30 mV. Participants were instructed to imitate the facial expressions they viewed. Each block lasted approximately 20 min.

## Results

### Confirmatory Analyses

Datasets are available in Supplementary Table S1. A difference value was calculated separately for happy, angry and neutral trials. This value was computed from the results of the Affectiva analysis and the EMG results using the following equations: (a) $\frac{j\,o\,y-a\,n\,g\,r\,y}{j\,o\,y+a\,n\,g\,r\,y}$, (b) $\frac{s\,m\,i\,l\,e-b\,r\,o\,w\,f\,u\,r\,r\,o\,w}{s\,m\,i\,l\,e+b\,r\,o\,w\,f\,u\,r\,r\,o\,w}$ and (c) $\frac{Z\,y\,g\,o\,m\,a\,t\,i\,c\,u\,s\,a\,m\,p\,l\,i\,t\,u\,d\,e-C\,o\,r\,r\,u\,g\,a\,t\,o\,r\,a\,m\,p\,l\,i\,t\,u\,d\,e}{Z\,y\,g\,o\,m\,a\,t\,i\,c\,u\,s\,a\,m\,p\,l\,i\,t\,u\,d\,e+C\,o\,r\,r\,u\,g\,a\,t\,o\,r\,a\,m\,p\,l\,i\,t\,u\,d\,e}$. The difference scores were used, as the EMG and Affectiva measures were obtained in different units. The scores ensured that all measures were transformed to lie within a scale between −1 and 1, ensuring the comparability of EMG and Affectiva measures. The computation of these scores resulted in one value for the EMG measure, one value for the Affectiva measure of emotion and one value for the Affectiva measure of expression. *One-tailed tests* were used for all analyses as pre-registered, as hypotheses were directional; however, note that this did not make a difference for significance of results in the current study.

**FIGURE 1:**
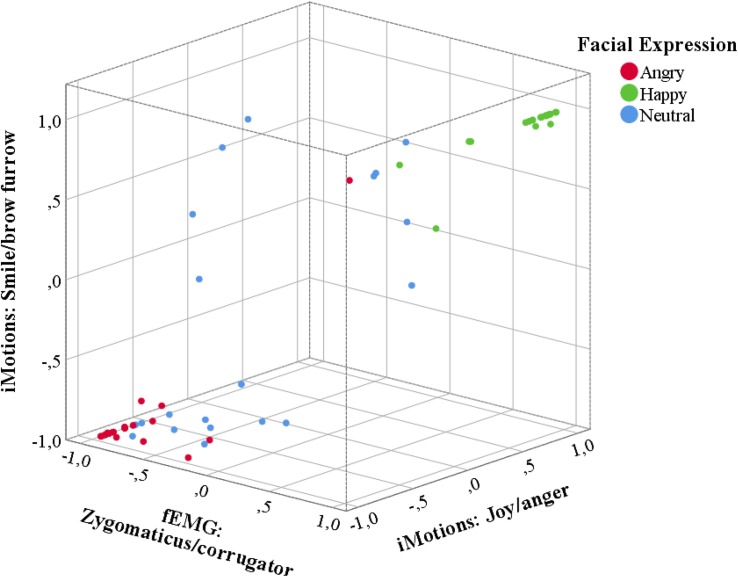
Three-dimensional scatter plot of mean difference scores between the Affectiva scores for smile and brow furrow and the difference scores between “joy” and “anger,” as well as between the EMG amplitudes for zygomaticus mayor and corrugator supercilii activity. Note that the difference score is computed to be more negative (closer to –1) if the respective measure indicates a more negative (i.e. angry) expression and more positive (closer to 1) if the measure indicated a positive (i.e. happy) expression. Red dots indicate the difference scores in the angry condition, green dots in the happy condition and blue dots in the neutral condition. Difference scores of all three types were significantly positive (close to 1) in the happy condition and significantly negative (close to –1) in the angry condition.

Correlations between the Affectiva difference score values and the EMG difference score were computed. Both of these values range from a scale between −1, indicating an angry expression to +1, indicating a happy expression. There was a significant correlation of the EMG measures with the difference score for Affectiva smile and brow furrow values, *r*(60) = 0.761, *p* < 0.001, and the difference score for Affectiva “joy” and “anger” values, *r*(60) = 0.789, *p* < 0.001 (Figure 1).

Furthermore, for each expression condition (happy, angry, neutral) separate one-sample-*t*-tests were computed to investigate whether each of the difference scores (joy/angry; smile/brow furrow, zygomaticus mayor/corrugator supercilii) differs from zero. Regarding the Affectiva Software, difference scores were significantly positive in the happy condition for both the smile/brow furrow, *M* = 0.97, *CI* = [0.92, 1.02], *t*(19) = 40.81, *p* < 0.001 and the joy/anger score, *M* = 1.00, *C*I = [0.9996, 1.00], *t*(19) = 8837.84, *p* < 0.001. They were significantly negative in the angry condition, for both the smile/brow furrow, *M* = −0.88, *CI* = [−1.08, −0.68], *t*(19) = −9.13, *p* < 0.001 and the joy/anger score, *M* = −0.80, *CI* = [−1.00; −0.59], *t*(19) = −8.25, *p* < 0.001. They did not differ from zero in the neutral condition for both the smile/brow furrow, *M* = −0.24, *CI* = [−0.64, 0.16], *t*(19) = −1.24, *p* = 0.116 and the joy/anger score, *M* = −0.24, *CI* = [−0.57, 0.08], *t*(19) = −1.55, *p* = 0.069. Regarding EMG, scores were also significantly positive in the happy, *M* = 0.63, *CI* = [0.44, 0.81], *t*(19) = 7.08, *p* < 0.001, and significantly negative in the angry condition, *M* = −0.79, *CI* = [−0.83; −0.74], *t*(19) = −34.41, *p* < 0.001, but they were also significantly negative in the neutral condition, *M* = −0.43, *CI* = [−0.55, −0.32], *t*(19) = −7.71, *p* < 0.001.

Following a reviewer suggestion, non-preregistered repeated measure ANOVAs were conducted, showing that mean Affectiva scores and mean EMG amplitudes were significantly different between the three conditions (all Greenhouse-Geisser corrected). Affectiva score “smile”: *F*(2, 38) = 151.07, *p* < 0.001, η*p*^2^ = 0.89, Affectiva score “brow furrow”: *F*(2, 38) = 31.65, *p* < 0.001, η*p*^2^ = 0.63, Affectiva score “joy”: *F*(2, 38) = 121.73, *p* < 0.001, η*p*^2^ = 0.87, Affectiva score “anger”: *F*(2, 38) = 20.9, *p* < 0.001, η*p*^2^ = 0.52, EMG zygomaticus mayor amplitude: *F*(2, 38) = 38.93, *p* < 0.001, η*p*^2^ = 0.67, EMG corrugator supercilii amplitude: *F*(2, 38) = 26.75, *p* < 0.001, η*p*^2^ = 0.59. Descriptive statistics are displayed in Table 1, showing that the Affectiva scores for joy and smile, as well as the EMG amplitudes of the zygomaticus mayor were highest in the happy condition, while the Affectiva scores for anger and brow furrow and the corrugator supercilii amplitude were highest in the angry condition. The pre-registered dependent sample *t*-tests (specifically addressing the hypotheses) confirmed these observations (Table 2). Note that the only exception from this pattern was the zygomaticus mayor score which was also significantly higher in the angry than the neutral condition.

**TABLE 1:** Descriptive statistics for the different outcome measures.

Measure		Condition	*M*	SD	Lower CI	Upper CI
Affectiva	Anger	Happy	0.00	0.00	0.00	0.00
		Angry	8.88	8.72	4.79	12.96
		Neutral	0.11	0.39	–0.07	0.29
	Brow furrow	Happy	0.60	1.82	–0.25	1.45
		Angry	36.72	29.52	22.90	50.53
		Neutral	1.13	3.42	–0.47	2.73
	Joy	Happy	67.53	27.29	54.75	80.30
		Angry	0.14	0.40	–0.05	0.32
		Neutral	0.20	0.56	–0.07	0.46
	Smile	Happy	69.56	25.20	57.77	81.36
		Angry	0.52	1.42	–0.14	1.19
		Neutral	0.34	0.86	–0.06	0.75
Affectiva (during EMG)	Anger	Happy	0.06	0.23	–0.04	0.17
		Angry	5.30	6.92	2.06	8.54
		Neutral	1.12	3.74	–0.63	2.87
	Brow furrow	Happy	7.35	17.46	–0.82	15.52
		Angry	30.39	27.64	17.45	43.33
		Neutral	8.96	21.42	–1.06	18.99
	Joy	Happy	75.59	16.54	67.85	83.33
		Angry	0.54	1.86	–0.33	1.41
		Neutral	1.46	4.89	–0.83	3.75
	Smile	Happy	77.17	16.71	69.35	84.99
		Angry	1.84	3.62	0.15	3.54
		Neutral	2.31	6.65	–0.80	5.42
EMG	Zygomaticus mayor	Happy	35.18	22.72	24.55	45.81
		Angry	4.48	3.22	2.97	5.99
		Neutral	2.43	0.78	2.07	2.80
	Corrugator supercilii	Happy	6.18	8.84	2.05	10.32
		Angry	42.41	30.00	28.37	56.45
		Neutral	7.32	4.15	5.38	9.26

**TABLE 2:** Dependent sample *t*-tests comparing the outcome measures between conditions.

Measure	Conditions	*t*-Value	df	*p*-Value	*p*-Value (one-tailed)	Cohen’s *d*
Corrugator supercilii	Happy-Angry	–5.44	19	0.000	0.000	–1.22
	Happy-Neutral	–0.49	19	0.629	0.315	–0.11
	Angry-Neutral	5.20	19	0.000	0.000	1.16
Zygomaticus mayor	Happy-Angry	6.03	19	0.000	0.000	1.35
	Happy-Neutral	6.49	19	0.000	0.000	1.45
	Angry-Neutral	2.96	19	0.008	0.004	0.66
Smile	Happy-Angry	12.23	19	0.000	0.000	2.73
	Happy-Neutral	12.38	19	0.000	0.000	2.77
	Angry-Neutral	0.46	19	0.648	0.324	0.10
Brow furrow	Happy-Angry	–5.60	19	0.000	0.000	–1.25
	Happy-Neutral	–1.45	19	0.163	0.081	–0.32
	Angry-Neutral	5.66	19	0.000	0.000	1.27
Joy	Happy-Angry	10.99	19	0.000	0.000	2.46
	Happy-Neutral	11.09	19	0.000	0.000	2.48
	Angry-Neutral	–0.38	19	0.706	0.353	–0.08
Anger	Happy-Angry	–4.55	19	0.000	0.000	–1.02
	Happy-Neutral	–1.27	19	0.221	0.111	–0.28
	Angry-Neutral	4.60	19	0.000	0.000	1.03

### Exploratory Analyses

To explore the reliability of the Affectiva software in trials, in which participants were wearing the electrodes, identical analyses were conducted with the difference scores of Affectiva Software computed during the EMG session. This exploratory analysis was conducted, firstly, to investigate whether both measures can be collected simultaneously, in case researchers want to combine the high sensitivity of EMG with the wide variety of different emotions recognized by Affectiva. Secondly, a direct within trial comparison of both measures was possible this way, using identical trials and therefore excluding the possibility of random variations in emotional expressions between trials.

**FIGURE 2:**
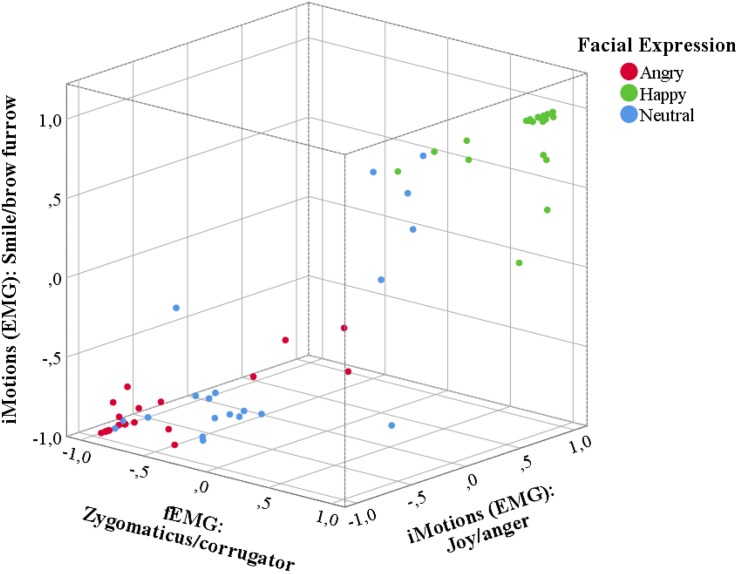
Three-dimensional scatter plot of mean difference scores between the Affectiva scores for smile and brow furrow and the scores for “joy” and “anger” during the EMG condition, as well as the EMG amplitudes for zygomaticus mayor and corrugator supercilii activity. Note that the difference score is computed to be more negative (closer to –1) if the respective measure indicates a more negative (i.e., angry) expression and more positive (closer to 1) if the measure indicated a positive (i.e., happy) expression. Red dots indicate the difference scores in the angry condition, green dots in the happy condition and blue dots in the neutral condition.

Correlations between the Affectiva values and the EMG were computed, showing a significant correlation of the EMG measures with the difference score for Affectiva smile and brow furrow values, *r*(60) = 0.754, *p* < 0.001, and the difference score for Affectiva joy and anger values, *r*(60) = 0.686, *p* < 0.001 (Figure 2).

One-sample *t*-tests showed that difference scores were still significantly positive in the happy condition for both the smile/brow furrow, *M* = 0.88, *CI* = [0.77, 0.99], *t*(19) = 16.69, *p* < 0.001 and the joy/anger score, *M* = 1.00, *CI* = [1.00, 1.00], *t*(19) = 667.11, *p* < 0.001. They were significantly negative in the angry condition, for both the smile/brow furrow, *M* = −0.80, *CI* = [−0.94, −0.65], *t*(19) = −11.43, *p* < 0.001, and the joy/anger score, *M* = −0.62, *CI* = [−0.92; −0.32], *t*(19) = −4.36, *p* < 0.001. However, there now was a significant difference from zero in the neutral condition for the smile/brow furrow, *M* = −0.48, *CI* = [−0.82, −0.13], *t*(19) = −2.88, *p* = 0.005, though not the joy/anger score, *M* = −0.17, *CI* = [−0.54, 0.20], *t*(19) = −0.96, *p* = 0.176. Absolute value differences between conditions were investigated using dependent sample *t*-tests, revealing the same pattern as for the Affectiva scores without simultaneous EMG recording (Table 3).

**TABLE 3:** Dependent sample *t*-tests comparing the Affectiva Scores during EMG testing between conditions.

Measure	Conditions	*t*-Value	df	*p*-Value	*p*-Value (one-tailed)	Cohen’s *d*
Smile	Happy-Angry	20.23	19	0.000	0.000	4.52
	Happy-Neutral	19.07	19	0.000	0.000	4.26
	Angry-Neutral	–0.45	19	0.658	0.329	–0.10
Brow furrow	Happy-Angry	–4.56	19	0.000	0.000	–1.02
	Happy-Neutral	–1.52	19	0.144	0.072	–0.34
	Angry-Neutral	4.07	19	0.001	0.000	0.91
Joy	Happy-Angry	20.39	19	0.000	0.000	4.56
	Happy-Neutral	19.64	19	0.000	0.000	4.39
	Angry-Neutral	–1.31	19	0.205	0.102	–0.29
Anger	Happy-Angry	–3.44	19	0.003	0.001	–0.77
	Happy-Neutral	–1.35	19	0.194	0.097	–0.30
	Angry-Neutral	3.07	19	0.006	0.003	0.69

Finally, paired *t*-tests were computed to explore differences in mean difference scores between the three different methods (Affectiva, Affectiva with attached EMG electrodes and EMG amplitudes) within each of the three emotion conditions (happy, angry, neutral). These analyses revealed no significant differences between the DV scores of the two Affectiva methods (videos with- and without attached EMG electrodes) for any of the three conditions. Affectiva DV scores (both for the measures “Smile” and “Brow furrow”, as well as the measures “Joy” and “Anger”) were significantly higher than the EMG DV scores in the happy condition, suggesting that Affectiva indicates a stronger positive expression than EMG. No differences between the measures were significant in the angry or neutral condition (Table 4).

**TABLE 4:** Comparison of mean difference scores between the three different methods (Affectiva, Affectiva with attached EMG electrodes and EMG amplitudes) within each of the three emotion conditions (happy, angry, neutral).

Condition	DV measures	Methods	*t*-Value	df	*p*-Value	*p*-Value (one-tailed)	Cohen’s *d*
Happy	Smile/Brow furrow	Affectiva – Affectiva/EMG	1.51	19	0.148	0.074	0.34
	Smile/Brow furrow – Zygo/Curro	Affectiva – EMG	4.22	19	<0.001	<0.001	0.94
	Smile/Brow furrow – Zygo/Curro	Affectiva/EMG – EMG	2.33	19	0.031	0.016	0.52
	Joy/Anger	Affectiva – Affectiva/EMG	1.08	19	0.293	0.147	0.24
	Joy/Anger – Zygo/Curro	Affectiva – EMG	4.19	19	0.001	0.001	0.94
	Joy/Anger – Zygo/Curro	Affectiva/EMG – EMG	4.16	19	0.001	0.001	0.93
Angry	Smile/Brow furrow	Affectiva – Affectiva/EMG	–0.65	19	0.526	0.263	–0.14
	Smile/Brow furrow – Zygo/Curro	Affectiva – EMG	–0.94	19	0.358	0.179	–0.21
	Smile/Brow furrow – Zygo/Curro	Affectiva/EMG – EMG	–0.14	19	0.893	0.447	–0.03
	Joy/Anger	Affectiva – Affectiva/EMG	–1.05	19	0.309	0.155	–0.23
	Joy/Anger – Zygo/Curro	Affectiva – EMG	–0.09	19	0.93	0.465	–0.02
	Joy/Anger – Zygo/Curro	Affectiva/EMG – EMG	1.13	19	0.272	0.136	0.25
Neutral	Smile/Brow furrow	Affectiva – Affectiva/EMG	0.96	19	0.349	0.175	0.21
	Smile/Brow furrow – Zygo/Curro	Affectiva – EMG	1.01	19	0.324	0.162	0.23
	Smile/Brow furrow – Zygo/Curro	Affectiva/EMG – EMG	–0.24	19	0.812	0.406	–0.05
	Joy/Anger	Affectiva – Affectiva/EMG	–0.30	19	0.766	0.383	–0.07
	Joy/Anger – Zygo/Curro	Affectiva – EMG	1.20	19	0.245	0.123	0.27
	Joy/Anger – Zygo/Curro	Affectiva/EMG – EMG	1.39	19	0.181	0.091	0.31

## Discussion

The current study aimed at comparing the Affectiva software with EMG concerning the identification of the imitated emotions happiness and anger compared to neutral expressions. We expected measures of Affectiva scores to be comparable with EMG measures. In line with our hypotheses, there was a significant correlation between EMG and Affectiva measures. Difference scores were significantly positive in happy conditions for all outcome measures (Affectiva joy/anger and smile/brow furrow scores and EMG zygomaticus mayor/corrugator supercilii scores) and negative in angry conditions. Only in the neutral condition, EMG scores were significantly negative, indicating that there was more corrugator supercilii than zygomaticus mayor activity. Contrasts between conditions (happy, angry, neutral) in raw scores of the measures confirmed the expected findings, with the emotion that was measured with each respective measure scoring significantly higher than both other emotions, which in turn did not significantly differ from one another, as they scored generally low. The only exception was that the Zygomaticus amplitude was also higher in the negative than the neutral condition.

In addition, we explored whether Affectiva is still applicable even when participants are wearing electrodes. Correlations with EMG scores were again very high. Happy expressions showed significantly positive scores and angry expressions negative scores. However, as for the EMG measures, neutral expressions now received negative scores.

In summary, both EMG and Affectiva Software could correctly identify happy and angry emotions imitated by participants and differentiate them from neutral faces. High correlations show that both methods are generally comparable. Furthermore, our exploratory analysis demonstrated that Affectiva software can still be used on videos of participants wearing electrodes used for facial EMG recording. However, compared to the videos in which participants were not wearing electrodes, the software was now less accurate, considering neutral facial expressions as negative. A previous study correlating FaceReader scores with fMEG, also suggested a tendency of the software to recognize neutral faces as negative – in this case “sad” (Suhr, 2017). However, note that in the current study during the EMG session both EMG and the Affectiva Software considered the neutral expression as negative. Therefore, one alternative explanation is that subjects might have displayed more negative facial expressions in the EMG condition, leading to negative scores in neutral conditions for EMG and Affectiva. The same participants completed all conditions, excluding the possibility that inert features of their faces caused the effect. Furthermore, the order of blocks with and without EMG was counterbalanced, excluding the possibility of an order effect. Possibly, the electrodes applied according to standard procedures affect facial expressions, leading to the observed differences. For example, there might be additional tension in the face due to the electrodes placed on the cheeks. In this case, Affectiva software used on videos without electrodes would provide more reliable values than EMG.

The current study focused on the most commonly researched emotions – happiness and anger – by investigating zygomaticus mayor and corrugator supercilii responses. Future research could explore other emotions. Furthermore, the current study explicitly instructed participants to imitate emotional expressions. Therefore, the intensity of displayed emotions may have been particularly strong (highest level intensity on the Facial Action Coding System). However, Stöckli et al. (2018) found the Affectiva software to be less precise in identifying emotional expressions when expressed in natural contexts and therefore more subtle emotions were analyzed than when participants were explicitly asked to display emotions. In contrast, EMG is highly sensitive and can therefore also detect very subtle or implicit displays of emotions. Additional research could explore implicit expressions of emotion in response to stimuli, to investigate the suitability of Affectiva compared to EMG in recognizing more subtle realistic emotional expressions. Furthermore, it should be noted that participants were asked to imitate emotional expressions. They may therefore not be experiencing these emotions but rather just acting them out. This method was chosen because previous research suggests that participants find it easier to display emotional expressions when they see them (Korb et al., 2010; Otte et al., 2011). Furthermore, the use of KDEF stimuli ensured that the expressions in the current study are related to a stimulus set that is often used for research. However, posed facial expressions may differ from spontaneously and naturally elicited expressions of emotions and may even be independent of the actual emotions that a person is feeling (Fridlund, 2014). The posed expressions from the validated KDEF database may furthermore differ from the expressions that each individual participant may display when expressing a specific emotion. Therefore, more natural displays of emotions should be used in future investigations. These more natural displays could either involve asking participants to display a specific emotion on their own, although these natural emotions may be less pronounced than facial displays while participants view the specific emotional expression (Korb et al., 2010; Otte et al., 2011). Alternatively, emotions could be elicited naturally, for example by presenting emotion-inducing videos. This manipulation may lead to more natural, though less controlled, facial displays of emotion.

Participants in the current study were asked to keep their head in a specific location during the recording. As people tend to keep a constant distance to computer screens this behavior is fairly natural in the lab. However, if more realistic scenarios are studied in the real world, the position may play a role. The temporal resolution of EMG can be very high, while the resolution of software is defined by the technical configuration of the camera used. In the current study a sampling rate of 60 Hz was sufficient to measure emotional displays.

In conclusion, the current study showed that the Affectiva software can detect the emotions “happy” and “angry” from faces and distinguish them from neutral expressions. The determined values further significantly correlate with EMG measures, suggesting that both methods are comparable.

## Data Availability Statement

All datasets generated for this study are included in the article/Supplementary Material.

## Ethics Statement

The studies involving human participants were reviewed and approved by the Ethics Committee of the Institute of Psychology at the University of Göttingen. The participants provided their written informed consent to participate in this study.

## Author Contributions

LK was involved in the conception and design of the work, supervising the acquisition and data analysis, interpreting the data, drafting the manuscript, and revising it critically for important intellectual content. DF was involved in the design of the work, the acquisition, analysis and interpretation of the data, and revising the manuscript. AS was involved in the conception and design of the work, supervising the data analysis, interpreting the data, and revising the manuscript critically for important intellectual content.

## Conflict of Interest

The authors declare that the research was conducted in the absence of any commercial or financial relationships that could be construed as a potential conflict of interest.

## Footnotes

1 Note that the term “joy” is used to describe happy facial expressions in the Affectiva software. However, in definitions used for research purposes, joy may describe a more general feeling, while the term “happy” is more often used to describe facial expressions related to basic emotions.
